# Partial rupture of the left main bronchus with left lung atelectasis due to blunt chest injury: a case report

**DOI:** 10.3389/fmed.2024.1434772

**Published:** 2024-07-25

**Authors:** Taiyu Bi, Xiaotian Duan

**Affiliations:** ^1^Thoracic Surgery, First Hospital of Jilin University, Changchun, China; ^2^Department of Nursing, First Hospital of Jilin University, Changchun, China

**Keywords:** blunt chest injury, bronchial rupture, trauma, pulmonary atelectasis, bronchoscopy

## Abstract

**Background:**

Bronchial injury is rare in blunt chest trauma, but can be life-threatening. The symptoms of patients with complete bronchial rupture are typical, and most of them are diagnosed in a timely manner and treated with surgery. However, for those with partial rupture of the bronchus, the symptoms are mild, the imaging results are negative, the possibility of delayed diagnosis is high, and serious complications can occur. Early diagnosis and treatment are key to treating this disease.

**Case description:**

We report a 52-year-old woman with mild mediastinal emphysema after blunt chest trauma. Left whole-lung atelectasis appeared after a period of conservative treatment. Bronchoscopy revealed injury of the left main bronchus, and surgery was performed. The patient’s lungs recovered well after surgery. CT (Computed tomography) examination during follow-up revealed that the structure of the left main bronchus was intact and unobstructed. The left lung was well recovered.

**Conclusion:**

For patients with mild symptoms of blunt chest trauma, mediastinal emphysema and subcutaneous emphysema; no pleural effusion or pneumothorax; and a negative chest CT, laryngoscopy or bronchoscopy should be performed in a timely manner to ensure the stability of the respiratory and circulatory system and confirm whether there is tracheobronchial injury. Surgical treatment should be performed in a timely manner after localization and diagnosis.

## Introduction

Bronchial injury is rare in patients with blunt chest trauma, with an incidence ranging from 0.8 to 5%. It often presents as partial or complete rupture of the bronchus, occurring within 2–3 cm from the tracheal carina, most commonly on the right side. The clinical symptoms are progressive subcutaneous emphysema, tension pneumothorax, massive leakage of air in the thoracic drainage tube. It is often complicated by rib fracture, hemothorax and respiratory failure. If not diagnosed and treated in time, the patient’s life will be threatened (1). Early diagnosis is very important for patients. The diagnostic methods used were emergency CT or bronchoscopy. Patients with complete tracheal rupture can be diagnosed with comprehensive imaging and clinical symptoms. However, the diagnosis of partial rupture is relatively difficult, and because of the lack of typical clinical symptoms and imaging findings, diagnosis is often delayed, resulting in complications. We report a case of delayed diagnosis of partial rupture of the left main bronchus.

## Case introduction

**Figure 1:**
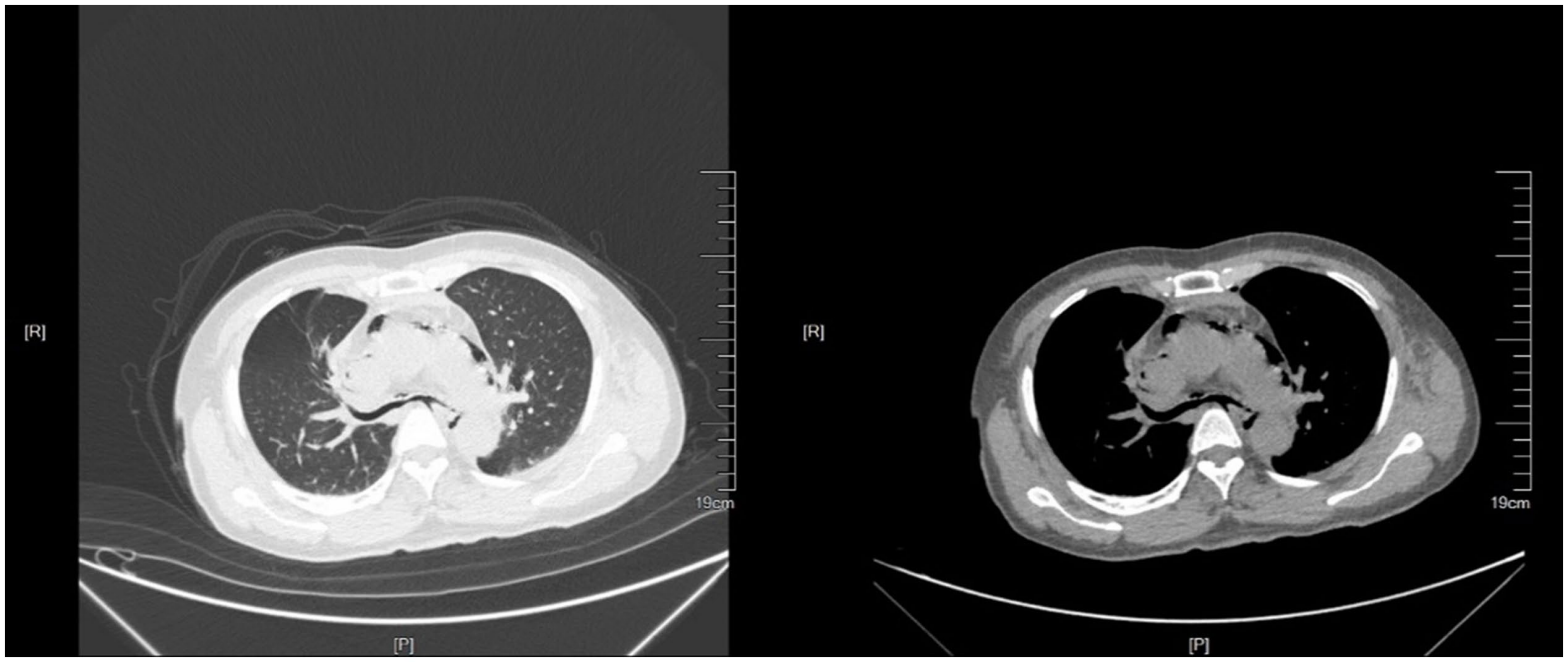
Chest CT at the first visit.

A 52-year-old female underwent blunt chest trauma after being crushed on her torso by a large machine. She was admitted to the emergency department 2 h after the injury. Her symptoms were chest pain, neck pain, and dyspnea. Her blood pressure was 140/80 mmHg, her heart rate was 100 beats/min, and her oxygen saturation was 95%. Physical examination revealed bilateral thorax symmetry, complete structure, ecchymosis on the front chest wall, soft tissue contusion of the chest wall caused by external force, a positive thoracic compression test, and a small amount of subcutaneous emphysema in the neck. Abdominal CT and cardiac B-mode ultrasound showed no significant abnormalities. Chest CT (Figure 1) revealed mediastinal emphysema, multiple rib fractures on both sides, esophagus and bronchus continuously visible, bilateral thorax without pneumothorax or hydropneumothorax. Head CT revealed bilateral parapharyngeal space, air accumulation in the posterior nasopharyngeal top and subcutaneous soft tissue. She was treated nonsurgically with chest strap fixation and anti-inflammatory, analgesic, and phlegm reduction agents. After 12 h of observation in the emergency department, the symptoms of chest pain and dyspnea were relieved, and the patient was discharged from the hospital.

**Figure 2:**
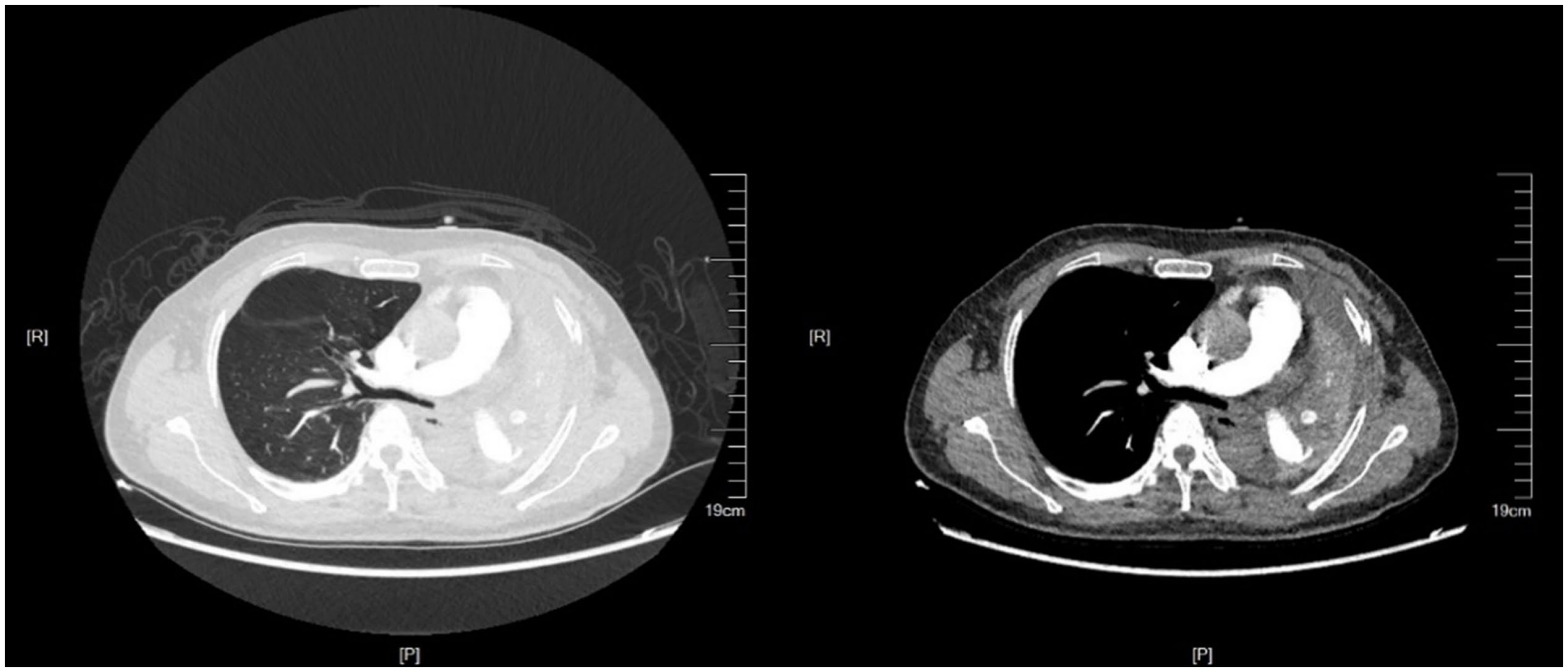
Chest CT at the second visit.

**Figure 3:**
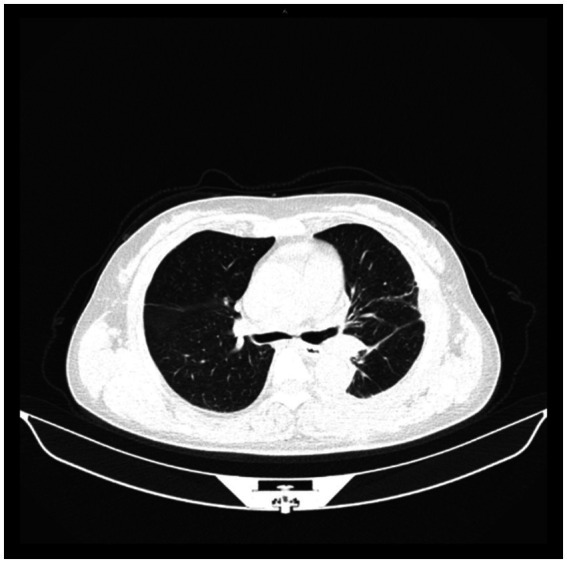
Chest CT at the time of reexamination after discharge from hospital.

Seventy days after her first visit, she was admitted to the thoracic surgery clinic, complaining of difficulty breathing and being unable to perform daily activities. Chest CT (Figure 2) revealed atelectasis of the left whole lung. Bronchoscopy revealed cicatricial occlusion of the left main bronchus (Figure 3). The patient was considered to have left main bronchus injury caused by blunt chest trauma, which led to atelectasis of the left whole lung. She underwent a thorax operation 3 days after admission. During the operation, a collapsed left lung with good color and blood flow was observed. After opening the mediastinal pleura, collapse of the left main bronchus 3 cm away from the carina was observed, and local scarring occurred. The patient was diagnosed with a partial rupture of the left main bronchus. Bronchial sleeve resection and bronchial end-to-end anastomosis were performed. The scar tissue of the left main bronchus was removed, continuous nontensioned end-to-end suturing was performed with 3-0 absorbable sutures, the patient was rinsed with normal saline after ventilation of both lungs, and no obvious air leakage was observed. After the suture was strengthened with 3-0 sutures, normal saline irrigation was performed again, and there was no obvious air leakage. A 28F drainage tube was placed at the top of the pleura, and a negative pressure drainage bucket was placed at the 7th intercostal space. The chest was closed layer by layer. Reexamination of chest radiographs and bronchoscopy 2 days after surgery showed that the left lung was well retained and that the left main bronchus was patent. She walked without fever or other adverse reactions. The drainage tube was removed after 3 days and the patient was discharged after 7 days.

**Figure 4:**
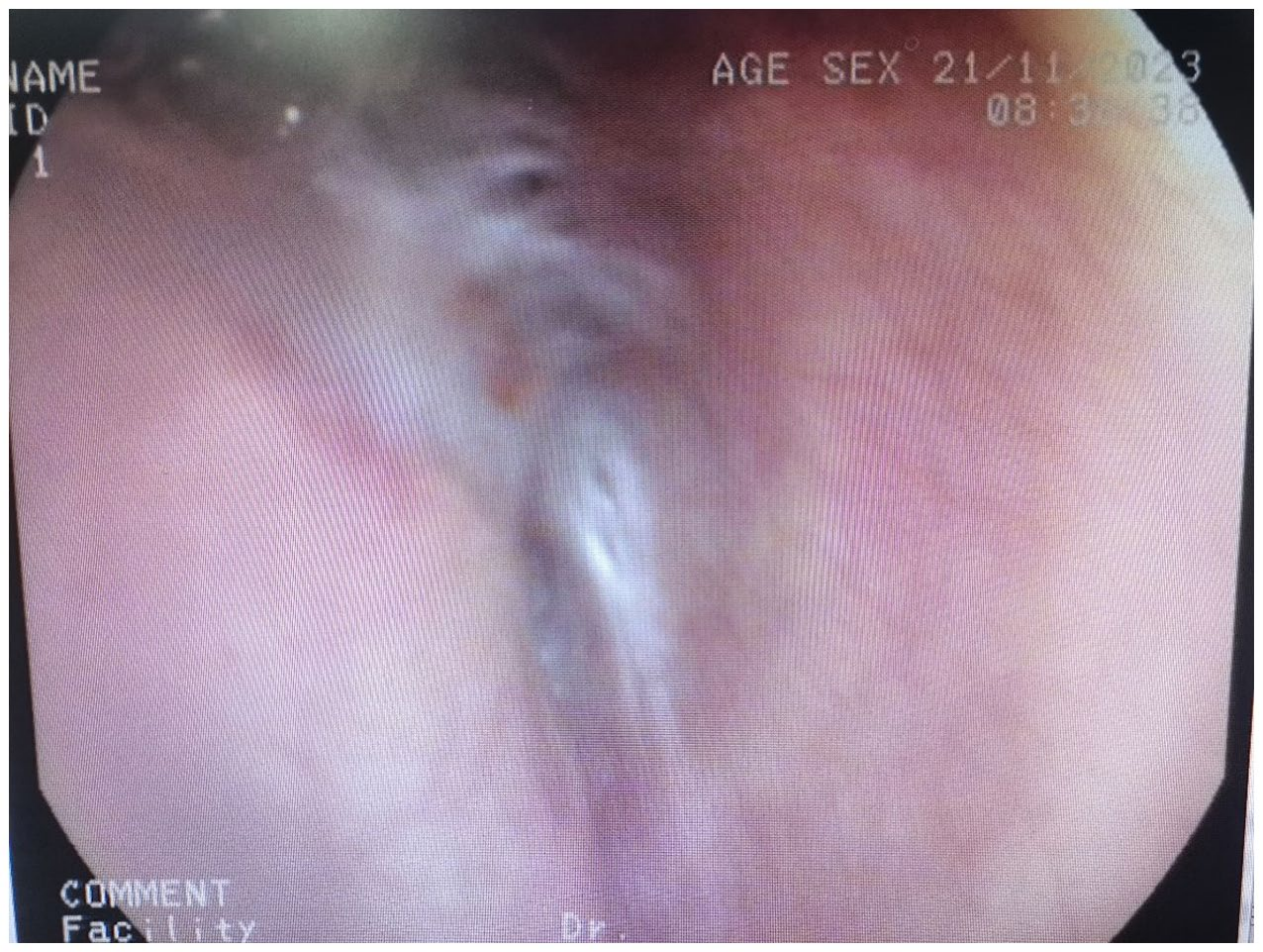
Left main bronchus seen under bronchoscope during the second visit.

Thirty days after discharge, she came to the hospital for chest CT examination (Figure 4), which revealed that the left lung was well retained, the left main bronchus was structurally intact, and the continuity was smooth. She had no obvious discomfort, and her work and life returned to normal.

## Discussion

Krish et al. (2) proposed three mechanisms of tracheobronchial injury caused by blunt chest injury: (1) The transverse diameter increased, and the anterior and posterior diameters decreased after the thorax was subjected to trauma. The negative pressure in the chest caused the lungs to contact the pleura, causing the lungs to separate laterally, and the traction force at the bulge was greater than the elasticity of the trachea itself, resulting in rupture. (2) When the chest is subjected to external force, the glottis is closed, the internal pressure of the bronchus increases abruptly after the bronchus is squeezed, and the thicker bronchus experiences greater tension, which exceeds its own elasticity and leads to rupture. (3) The rapid deceleration of bronchial motion results in shear force, which acts on the cricoid cartilage and protuberance, resulting in injury. This case was caused by the first two causes, resulting in local bronchial rupture, a small amount of mediastinal emphysema and subcutaneous emphysema, a negative chest CT, and no obvious symptoms.

Bronchial injury is characterized by partial or complete rupture of the bronchus. Clinical symptoms vary according to the degree and location of injury. If complete airway rupture occurs outside the mediastinum and communicates with the pleural cavity, imaging can reveal a clear sign of a hilar fall, a large amount of pneumothorax (3). Continuous air leakage can be observed in patients who undergo closed thoracic drainage, and there is no relief. If the fracture occurs in the mediastinum and is not the same as that in the pleural cavity, the fractured bronchus may release a large amount of gas, resulting in a large amount of mediastinal emphysema and subcutaneous emphysema, and gas compression of the airway results in obvious dyspnea (4). Both of these conditions can be life-threatening. However, if the bronchus is partially broken, and the tear is located in the mediastinum, there is often less mediastinal emphysema, and the symptoms of dyspnea are not obvious. Imaging only reveals a small amount of mediastinal emphysema, which needs to be distinguished from simple mediastinal emphysema. In the process of postural movement or self-compensation, the soft tissue covers the ruptured mouth, the patient’s symptoms can be relieved, and it is easy to miss the diagnosis. During the process of chronic spontaneous healing, scar formation occurs, and whole-lung atelectasis occurs. Because the left main bronchus is more protected by peripheral blood vessels and tissues than the right side, its length is longer than that of the right side. In blunt injury, injury to the left side is less common than injury to the right side, the death rate is low, the symptoms are mild, and the diagnosis is not easy (5).

Demondion et al. (6) reported the case of a 7-year-old boy who was not fit for hospital treatment 6 months after trauma and was diagnosed by CT as atelectasis of the left whole lung and hyperventilation of the right lung; the left main bronchus was transected 1 cm away from the carina. Odell et al. (7) reported the case of a 28-year-old man with multiple injuries who received closed thoracic drainage on admission. Fifty-six days after the injury, dyspnea suddenly appeared during rehabilitation after the fracture reduction and fixation surgery. CT was used to diagnose atelectasis of the left whole lung and stenosis of the left main bronchus, and transection of the left main bronchus 4 cm from the carina was investigated during the operation. Samples et al. (8) reported a 29-year-old woman with a traumatic aortic valve tear combined with rupture of the left main bronchus. Fourteen days after the injury, CT revealed occlusion of the left main bronchus at 1 cm from the carina, and local scarring was explored intraoperatively. Ozcelik et al. (9) reported a 15-year-old female with right whole -lung atelectasis and pleural effusion found 75 days after injury who was diagnosed by surgical exploration as having local scar formation with chylothorax due to rupture of the right main bronchus. The above cases were caused by blunt chest injury, and the bronchial anatomy was reconstructed by surgery. Similar to our case.

Bronchoscopy is the most reliable and preferred method for confirming the location, depth and extent of bronchial rupture (2). We believe that the possibility of bronchial injury should be considered in patients with blunt chest injury. High suspicion and prompt diagnosis are key. The patient had a history of blunt chest trauma with mild symptoms, mediastinal emphysema and subcutaneous emphysema, no pleural effusion or pleural pneumothorax, closed thoracic drainage was not performed, and chest CT examination was negative. On the premise that the function of the respiratory and circulatory system is stable, laryngoscopy or bronchoscopy should be performed in a timely manner to confirm whether there is bronchial injury, locate and diagnose the injury, and perform timely surgical repair. Patients with atelectasis and loss of lung function were avoided.

At present, there is no clear consensus on the indications for conservative treatment of bronchial injury (1). Cardillo et al. (10) proposed a morphological classification of grade I to IIIB injuries for bronchial injuries. They concluded that surgery was primarily appropriate for Grade IIIB traumatic bronchial branch airway injuries. This is a class IIIA injury. In their study, two patients with Grade IIIA injuries were cured with conservative treatment. However, after this routine conservative treatment, local scarring resulted in atelectasis of the left whole lung. This indicates that the indications for conservative treatment should be considered with great caution, and if conservative treatment fails, the subsequent operation will be very unfavorable (11). Therefore, we believe that emergency surgery should be performed to reconstruct the trachea after early diagnosis to avoid serious complications. Conservative treatment may be considered if the tear is less than 2 cm or 1/3 of its diameter, vital signs are stable, spontaneous breathing is possible, and there are no clear signs of mediastinitis or sepsis (5). When conservative treatment is not satisfactory, surgery is needed. This requires additional case verification.

In patients with complications such as atelectasis, a thoracotomy may be performed to determine the surgical option (pneumonectomy or bronchoplasty). This depends on damage to the lung at the time of exploration. In this case, it takes 1–3 weeks to maintain normal ventilation due to early granulation tissue formation until the bronchus is blocked. During this period, lung tissue is mostly uninfected, providing conditions for reconstruction (9). This patient had good pulmonary blood supply and no clear signs of infection. The patient recovered well after reconstruction.

## Conclusion

The key to the treatment of bronchial injury caused by blunt chest injury is early and clear diagnosis and treatment, and the diagnosis of these patients should adopt the principle of “no missed diagnosis.” For patients with mild symptoms accompanied by mediastinal emphysema and subcutaneous emphysema, without pleural effusion and pleural gas, without closed thoracic drainage, unable to observe air leakage, and with a negative chest CT, laryngoscopy or bronchoscopy should be performed in a timely manner to confirm whether there is tracheobronchial injury after respiratory and circulatory system function is stable, and surgical repair should be performed after the location is confirmed. A small number of patients have indications for conservative management, which needs to be confirmed in more patients. Complications such as atelectasis should be treated with surgery, and the patient’s lung function should be preserved as much as possible. At the same time, regular follow-up is also the key to ensuring efficacy.

## Data availability statement

The original contributions presented in the study are included in the article, further inquiries can be directed to the corresponding author.

## Ethics statement

Written informed consent was obtained from the participant/patient(s) for the publication of this case report.

## Author contributions

TB: Writing – original draft, Writing – review & editing. XD: Writing – review & editing.
